# Experimental Studies on the Effect of Expired Amiodarone Drug (EAD) as a Corrosion Inhibitor on Mild Steel in 1 M HCl

**DOI:** 10.3390/ma17030751

**Published:** 2024-02-04

**Authors:** H. Mohamed Kasim Sheit, S. Musthafa Kani, M. Anwar Sathiq, S. S. Syed Abuthahir, P. Subhapriya, K. S. Nivedhitha, M. A. Umarfarooq, Irfan Anjum Badruddin, Sarfaraz Kamangar, Abdul Saddique Shaik

**Affiliations:** 1 PG and Research, Department of Chemistry, Jamal Mohamed College (Autonomous), Affiliated to Bharathidasan University, Tiruchirappalli 620020, Tamil Nadu, India; kasimchem1985@gmail.com (H.M.K.S.); musthafkani32@gmail.com (S.M.K.); syedchemjmc@gmail.com (S.S.S.A.); 2 Department of Chemistry, Bannari Amman Institute of Technology (Autonomous), Erode 638401, Tamil Nadu, India; subhapriyachem@gmail.com; 3 Centre for Material Science, School of Mechanical Engineering, KLE Technological University, Hubballi 580031, Karnataka, India; k.s.nivedhitha@gmail.com (K.S.N.); umarfarooq.ma@gmail.com (M.A.U.); 4 Mechanical Engineering Department, College of Engineering, King Khalid University, Abha 61421, Saudi Arabia; irfan@kku.edu.sa (I.A.B.); ssaheb@kku.edu.sa (S.K.); ashaik@kku.edu.sa (A.S.S.)

**Keywords:** amiodarone drug, HCl medium, mild steel, adsorption isotherm, corrosion, inhibitor

## Abstract

In the present investigation, the corrosion tendency of mild steel under acidic pH was studied by employing unused expired amiodarone (EAD) drug as a potential corrosion inhibitor by adopting the weight loss measurement method. The corrosion inhibition efficiency (IE) of the formed protective film (EAD) on the steel surface was analyzed using potentiodynamic polarization and AC-impedance spectroscopy studies. The surface morphology of the mild steel before and after corrosion (in 1.0 M HCl) was analyzed via scanning electron microscopy–energy dispersive X-ray spectroscopy (SEM–EDAX), atomic force microscopy (AFM), and thermodynamic studies. The weight loss measurement under different concentrations of EAD indicated that an excellent inhibition was displayed at a concentration of 0.001 M, and the IE was found to depend on both the concentration and molecular structure of EAD. A potentiodynamic polarization study revealed that EAD predominantly acted as a cathode inhibitor, and electrochemical impedance spectroscopy (EIS) confirmed the adsorption of EAD on the surface of mild steel, which obeyed Temkin’s adsorption isotherm model. The calculated thermodynamic parameters revealed that adsorption was spontaneous and exothermic.

## 1. Introduction

In the industrial era, mild steel finds widespread uses in several industries such as the petroleum, chemical, marine, automotive, aerospace, and construction sectors, etc., for the fabrication of goods and structures. Mild steel exhibits substantial corrosion susceptibility when subjected to corrosive agents during industrial procedures [1,2,3,4,5]. Corrosion is the chemical phenomenon wherein a metal’s surface undergoes degradation as a consequence of its interaction with the surrounding environment. It may arise from the metal undergoing interaction with a liquid or gaseous substance. Due to its deleterious and hazardous effects, it may lead to a fiscal detriment. Various methodologies have been proposed to mitigate this form of detriment [6,7]. Multiple investigations have substantiated the utilization of pharmaceutical agents for corrosion mitigation as an ecologically conscientious approach, devoid of any deleterious repercussions [8,9]. Pharmaceutical compounds possess the capability to function as adversaries to green corrosion inhibitors [10]. The vast majority of products can be synthesized utilizing abundant natural resources. In the realm of drug structures, the prevalence of carbocyclic or heterocyclic systems was notably pronounced. The selection of the most suitable pharmaceutical agent for the mitigation of corrosion was impacted by a multitude of factors [11,12,13]. To efficiently counteract corrosion, it is crucial to incorporate elements such as sulfur, oxygen, and nitrogen with drugs. The identification of an optimal pharmaceutical agent for corrosion mitigation can be expedited by taking into account two pivotal variables: the molecular dimensions and the solubility characteristics of the compound [13]. While it is widely accepted that the majority of pharmaceuticals maintain their efficacy beyond their expiration date, their application for purposes other than their designated use is restricted by liability and professional limitations. Recent studies have evaluated the effectiveness of past-expiration-date medications as corrosion inhibitors. Therefore, using old medications as corrosion inhibitors helps with the unused organization of old drugs and cuts down on financial losses. For instance, an expired atorvastatin drug was used as a corrosion inhibitor for mild steel in a hydrochloric acid solution [14]. Expired and unused carbamazepine and paracetamol tablets were used as corrosion inhibitors [15]. The former exhibited corrosion inhibition on carbon steel in 0.1 mol L^−1^ sulfuric acid solution to an extent of 90%, whereas the latter offered corrosion IE in acetic acid (0.25 mol L^−1^) to the range of 95%. Hence, in the present study, an attempt has been made to employ unused EAD as an inhibitor for 1 M HCL. EAD belongs to the class III category according to Vaughan Williams’ classification and serves as an antiarrhythmic agent. Amiodarone plays a prominent role in the therapeutic management of both acute and chronic ventricular and supraventricular arrhythmias. This investigation involved exploring its efficacy in mitigating attrition of mild steel in 1 M HCl solution at different temperature. The present work was focused on the evaluation of the IE of EAD on mild steel via the weight loss method, potentiodynamic polarization analysis, and electrochemical impedance measurements. This study was also focused on the evaluation of the surface morphology of mild steel using scanning electron microscopy–energy dispersive spectroscopy (SEM–EDAX) and atomic force microscopy (AFM). The specimen and solution preparations and instrumentation details are discussed under experimental techniques and the findings are presented in the results and discussion section.

## 2. Experimental Techniques

### 2.1. Preparation of Specimen

A mild steel specimen (1.0 × 4.0 × 0.2 cm^2^) was polished to a mirror finish, degreased with acetone, and used for a weight loss study. In addition, a 1 cm^2^ size specimen area was used for potentiodynamic polarization, electrochemical impedance spectroscopy, SEM, and AFM investigation.

### 2.2. Preparation of Solution

The acid solution (1.0 M HCl) was prepared via dilution of analytical grade HCl with double distilled water. All tests were conducted at different temperatures under mechanical stirring. The expired amiodarone (EAD) inhibitor stock solution was prepared by dissolving it in ethanol [16,17,18,19,20,21].

### 2.3. Determination of Corrosion Rate (CR) via Weight Loss Method

The weight of the specimens was recorded before they were immersed in a test solution containing 100 mL in a beaker, both with and without the presence of an inhibitor. After conducting a preliminary experiment on weight loss, several doses of EAD inhibitors ranging from 0.0000001 M to 0.001 M were tested. The samples were submerged in each test solution for two hours. After two hours, the samples underwent a series of procedures including cleaning, drying, and subsequent reweighing, which were repeated seven times. The IE was expressed in a percentage, and the corrosion rate, measured in W, was determined using the following mathematical formulae:

(1) $$w=\frac{\left(W_{1}-W_{2}\right)}{St}$$

(2) $$IE\;\%=\;[\frac{\left(W_{1}-W_{2}\right)}{W_{1}}]\;\times \;100$$

where *S* = total specimen surface area, *t* = the immersion time, and *W*_1_ and *W*_2_ were the initial and final specimen weights, respectively [22,23].

For measuring the CR, specimens were meticulously quantified and suspended employing glass hooks. The specimens were submerged in 100 mL of distilled water, accompanied by varying quantities of the EAD inhibitor, over 24 h. This process was replicated utilizing both immersion and non-immersion techniques. Following the completion of the 2-h duration, specimens were taken out and rinsed using fresh water, subsequently subjected to desiccation, and subsequently re-evaluated in terms of their mass. The corrosion rates were determined by quantifying the disparity in mass of each specimen utilizing the equation [24]

(3) $$CR=\frac{83.67\times w\;(mg)}{A\times T\times D}mmy$$

where A = area, T = time and D = density, w = weight loss (mg)

The chemical equation employed to determine the corrosion IE is stated below (4).

(4) $$IE\;\left(\%\right)=\left[1-\frac{W_{2}}{W_{1}}\right]\times 100$$

where *W*_1_ represents the corrosion rate without the inhibitor and *W*_2_ represents the corrosion rate with the inhibitor.

### 2.4. Specimen Surface Area Determination

The dimensions of mild steel, including its length, breadth, and thickness, were measured using Vernier calipers of high precision. In addition, the entire radii and surface areas of the specimens were determined. The samples were subjected to pre- and post-corrosion mass measurements. The masses of the specimens were determined before and after immersion utilizing a balance, namely the A meter balance-M5 type model [25].

### 2.5. Potentiodynamic Polarization Study

The polarization analysis was performed on three-terminal cells. The specimen was fabricated using a low-density metallic substrate, featuring a cathode surface area of 1 cm^2^ on one side, while the rest of the region was coated with a red enamel layer. A calomel cathode thoroughly saturated with liquid was employed, while a platinum foil with a quadrangular morphology was paced as an anode. The counter terminals experienced a notable environmental shift leading to the creation of a substantial cathode. The platinum cathode and activity terminal were immersed in distilled water to remove any inhibitory substances. The experimental procedure involved the utilization of a saline and saturated calomel cathode. The correlation between the *E* value and the logarithm of the current (*I_corr_*) was obtained from the plot; the electrochemical potential for consumption (*E_corr_*) and the Tafel slopes ‘ba’ and ‘bc’ were also predicted from the plot [26].

### 2.6. AC Impedance Measurements

The CHI-electrochemical workstation equipped with the impedance model 660A was used for the investigation of AC impedance. In terms of the experimental setting, there was no discernible difference between the cell setup and the polarization measurements. For this, a 10 mV sinusoidal potential was added on top of the steady-state potential by systematically shifting the AC frequency between 100 kHz and 100 MHz to calculate the real and imaginary components of the cell’s resistance (in ohms) at various frequencies. *R_t_* and *C_dl_*, or charge transfer resistance and double-layer capacitance, were calculated. *C_dl_* values were calculated with the help of the following formula [27,28]:

(5) $$C_{dl}=\frac{1}{2\times 3.14\times R_{t}\times f_{max}}$$

### 2.7. Surface Characterization Studies

The samples were immersed in a blank solution and EAD solutions for 2 h. After 2 h, the specimens were taken out and subjected to desiccation. The film molded on the surface was subjected to analysis using a variety of techniques to determine its chemical composition and properties.

### 2.8. Scanning Electron Microscopy (SEM)

The surface morphology of specimens before and after corrosion was analyzed using Hitachi, CAREL ZEISS EVO 18, Oberkochen, Germany. [29].

### 2.9. Energy Dispersive Analysis of X-rays (EDAX)

The sample was engrossed in a solution comprising an inhibitor for 24 h. Subsequently, it was extracted, cleansed using double distilled water, desiccated, and exposed to EDAX to scrutinize the constituents existing on the exterior of the specimen [30]. The investigation of the constituents existing on the exterior was performed utilizing the Bruker Nano computerized EDAX system, fabricated by Bruker Nano, GMBH, Berlin, Germany.

### 2.10. Atomic Force Microscopy Characterization (AFM) 

After being immersed in an inhibitor solution and another chemical for 24 h, a specimen made of mild steel was removed. It was then cleaned with double-distilled water, dried, and afterward examined for its surface properties [31]. The surface morphology of the specimen was examined using atomic force microscopy (AFM) using the Agilent Technologies (Santa Clara, CA, USA) 5500 series.

## 3. Results and Discussion

### 3.1. Weight Loss Method

The corrosion IE and corrosion rate (CR) under 1 M HCl solution were determined using the weight loss method and the obtained results are presented in Table 1.

The CR and IE were calculated for a blank and different concentrations of unused expired EAD inhibitors. This study involved measurement of CR and IE for the concentration ranged from 0.0000001 M to 0.001 M. It was observed that 0.001 M EAD solution had an inhibitory effectiveness of 88.77%. The increased corrosion efficiency was attributed to the incorporation of heteroatom on the metal surface of mild steel, which prevents corrosion. The results indicated that EAD offered good anti-corrosive properties. The findings of this investigation were in concordance with the outcomes of prior studies of other expired drugs [32,33,34,35].

### 3.2. Effect of Temperature

In a general sense, the phenomenon of corrosion exhibits an accelerated rate of manifestation when exposed to elevated temperatures. As the temperature increased, a concomitant rise in the corrosion rate of the specimen was observed, accompanied by a decrease in the effectiveness of corrosion prevention measures. At elevated temperatures, the solubility was augmented, and corrosion phenomena transpired with greater celerity. In electrochemical studies, the evaluation of temperature impact was crucial for understanding the adsorption mechanism of inhibitor molecules on the surface of the specimen. The temperature range from 303 K to 333 K was employed for the measurement of CR and IE [36]. The influence of the temperature on IE and CR were presented in Table 2.

The IE experienced a decrease from 88.77% to 34.70%, whereas the corrosion rate underwent an increase from 0.0941 to 0.5476, as the temperature rose from 303 K to 333 K in a 1 M HCl solution at the maximum concentration (0.001 M) for EAD. The rate at which inhibitor species desorbed from a metal surface and the temperature at which this occurred were strongly related to the concentration of the inhibitor present.

### 3.3. Adsorption Isotherm

The adsorption isotherm was a graphical representation that depicted the relationship between the extent of adsorption and the concentration of inhibitors. The electrochemical phenomenon occurring on the metal surface was expected to exhibit a strong correlation with the adsorption behavior of the inhibitor. The adsorption process was widely acknowledged to be dependent on the molecular configuration of the inhibitors. Adsorption isotherms afford valuable elucidation regarding the interplay between metal inhibitors. The adsorption isotherm serves as a pivotal tool in comprehending the interplay between the surface and the molecular constituents of an inhibitor [37].

When corrosion-inhibiting molecules come into contact with a steel surface, they displace water molecules already adsorbed there:

(6) $$M-H_{2}O+I↔M-I+H_{2}O$$

The kinetics of inhibitor molecule adsorption on the mild steel-solution interface were sensitive to a wide range of conditions. Among them were the environment’s temperature, chemical makeup, inhibitor concentration, and the interface’s electrochemical potential. Factors that affected how well an inhibitor bound to a metal included the inhibitor’s molecular dimensions, the inhibitor’s charge density, the strength of connections between the inhibitor and the metal, and the presence of metallic complexes. To investigate the adsorption of EAD on mild steel, several isotherm models have been proposed [38].

### 3.4. Temkin Adsorption Isotherm

**Figure 1:**
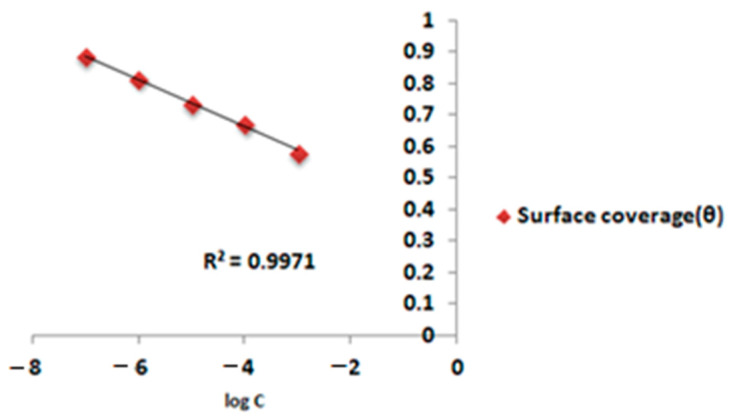
Temkin’s adsorption isotherm plot for the inhibition effect of EAD on mild steel in 1 M HCl at different temperatures.

The Temkin adsorption isotherm was created based on the postulation of a homogenous dispersion of the EAD inhibitor resulting in the development of a monolayer. The adsorption energy demonstrated a linear decline with increasing surface coverage values (θ). The ‘θ’ values were ascertained via weight loss measurements for the different concentrations of the EAD inhibitor in a 1 M HCl solution. These values were subjected to fitting using the Temkin adsorption isotherm, as illustrated in Figure 1, and the value of free energy change with respect to temperature is presented in Table 3.

(7) $$\exp\left(-2a\theta \right)=K_{ads}\times C\;\theta =-2.303\log K_{ads}/(2a-2.303\;logC)/2a$$

The “*a*” here stands for the lateral molecular interaction parameter [39]. The inhibitor molecule interacts with water molecules dwelling on the surface of the mild steel through a quasi-substitutional process, resulting in the adsorption phenomena.

(8) $${Org}_{sol}+{{xH}_{2}O}_{ads}\to {Org}_{ads}+{H_{2}O}_{sol}$$

In this context, *x* symbolizes the size parameter, denoting the quantity of adsorbed water molecules displaced by the provided adsorbate.

The coefficients (R^2^) for the EAD inhibitor system deviate somewhat from 0.9 at different temperatures but converge toward unity. Empirical research indicated a weak correlation between θ and log C in the EAD inhibitor system. R^2^ exceeds 0.9 for the inhibitor EAD system. Negative ${\Delta G}_{ads}^{o}$ values indicated the thermodynamic adsorption of inhibitor compounds from EAD onto mild steel surfaces. ΔG values below −20 kJ mol^−1^ favored physisorption, whereas values over −40 kJ favored chemisorption. The rise in *K_ads_* values and reduction in negative ${\Delta G}_{ads}^{o}$ values at 303–333 K indicated physisorption.

### 3.5. Free Energy of Adsorption and Energy of Activation Parameters for Anti-Corrosion Process

#### 3.5.1. Free Energy of Adsorption (${\Delta G}_{ads}^{o}$)

The ${\Delta G}_{ads}^{o}$, which represent the free energy of adsorption, were determined at different temperatures and 0.001 M concentrations using the Langmuir adsorption isotherm model. This computation was performed using Equation (9),

(9) $${\Delta G}_{ads}^{o}=-2.303RT\log\left(K_{ads}\times 55.55\right)$$

The determined average ${\Delta G}_{ads}^{o}$ values at several temperatures were given in Table 3.

The negative ${\Delta G}_{ads}^{o}$ values acquired through the Temkin’s adsorption isotherm indicated the spontaneous adsorption of inhibitor molecules on the steel surface. The ${\Delta G}_{ads}^{o}$ values exhibit a range of up to −20 kJ mol^−1^, which aligns with physisorption, while values exceeding −20 kJ mol^−1^ align with chemisorption [40].

#### 3.5.2. Energy of Activation (*E_a_*)

**Figure 2:**
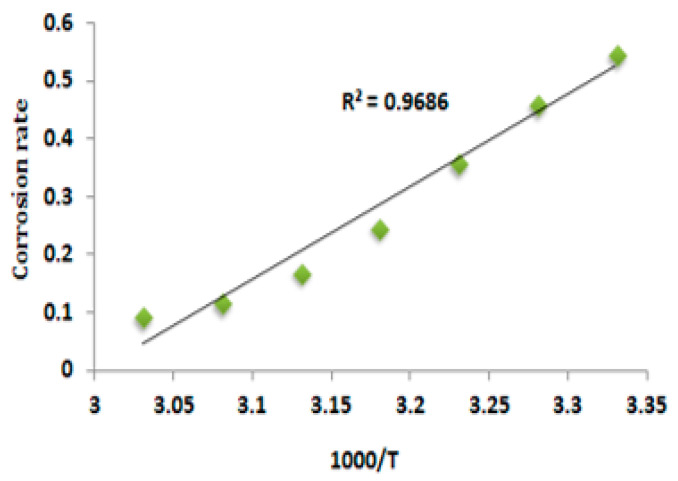
Arrhenius plot for the deterioration of mild steel in 1 M HCl solution with 0.001 M of EAD inhibitor.

The determination *E_a_* for the deterioration of mild steel immersed in a 1 M HCl solution was achieved by utilizing the Arrhenius model. The utilized equation in this context can be expressed as *log CR = Kexp^(−^^Ea^^/RT^*^)^; *R* represents the universal gas constant; *T* signifies the absolute temperature; and K represents the Arrhenius pre-exponential constant. The values of *E_a_* were determined for the mild steel when it was submerged in the EAD inhibitor system. The aforementioned values were derived from the gradient of the corrosion rate using Figure 2, and the calculated values ‘*E_a_*’ were given in Table 4.

The determined *E_a_* values exhibit an elevated magnitude when the concentration of the inhibitor was improved in contrast to the uninhibited system (blank). The observation of a lower *E_a_* at higher concentrations compared to the blank indicated a chemisorption mechanism, while the observation of a higher *E_a_* at higher concentrations compared to the blank suggested that a physical adsorption mechanism was followed during inhibitor action on mild steel [41].

At a temperature of 303 K, it has been observed that EAD exhibits enhanced efficacy. This can be attributed to the significantly higher activation energy (*E_a_*) values observed in the system with the maximum inhibitor concentration, as compared to the blank system. Thus, the diminishment of inhibition efficiencies was observed, leading to an escalation in the CR at elevated temperatures. The inhibitor was desorbing, exposing more of the surface to the air and other elements at high temperatures. At lower temperatures, the aforementioned problem was reduced due to the surface covering approaching saturation [42].

### 3.6. Electrochemical Investigation

The electrochemical method involves the measurement of the corrosion kinetics of mild steel. It makes it easy to gauge the efficiency of corrosion inhibitors based on the longevity of protective layers on surfaces and other factors in a short time. The following procedures were employed to determine if the specimen was in the lack or presence of EAD inhibitor, and an analysis showed that EAD exhibited cathode inhibition when immersed in a 1 M HCl solution. It is also used to provide a suitable mechanical framework for explaining corrosion prevention on mild steel when it is exposed to corrosive conditions.

#### 3.6.1. Potentiodynamic Polarization Study

**Figure 3:**
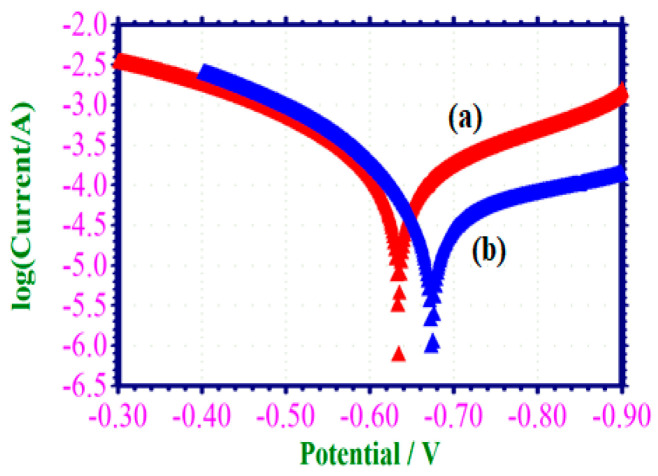
(a) Potentiodynamic polarization arcs for corrosion in 1 M HCl with and without EAD inhibitor mild steel in 1 M HCl (blank); (b) mild steel in 1 M HCl with 0.001 M of EAD.

The potentiodynamic polarization technique has been employed to scrutinize the development of a corrosion-preventing film on the surface of mild steel [43]. Upon the formation of a protective film, a discernible increase in the linear polarization resistance values (LPR) and a significant decrease in the corrosion current value *(I_corr_*) were observed. The PDP arcs of the specimen immersed in a solution containing 1 M HCl, both in the absence and presence of the electrochemical adsorption of inhibitor were illustrated in Figure 3. The corrosion parameters, namely the corrosion potential (*E_corr_*), Tafel slopes (bc and ba), linear polarization resistance (LPR), and corrosion current (*I_corr_*) were presented in Table 5.

Based on the data depicted in Figure 3, it was evident that when mild steel was subjected to submersion in a 1 M HCl solution, the corrosion potential was measured as −632 mV relative to the standard calomel electrode (SCE). The measured LPR (Linear Polarization Resistance) value was determined to be 291 Ω/cm^2^. The corrosion current demonstrated a magnitude of 1.2730 × 10^−4^ A/cm^2^. The corrosion potential demonstrated a noble ward shift, precisely at a magnitude of −672 mV/SCE. This observation revealed that the corrosion potential experiences a shift toward the less noble direction due to the formation of a protective layer on the surface of the mild steel. The formed protective film displayed a cathode inhibition tendency rather than anode inhibition behavior due to the formation of the Fe^2+^-EAD complex on the cathode region of mild steel [44,45]. This inhibitor was predominantly cathode type, as evidenced by its observed corrosion potential shift towards a less negative direction when compared to the blank value. The observed rise in LPR from 291 to 792 and the corresponding decline in *I_corr_* values from 1.2730 × 10^−4^ to 4.6340 × 10^−5^ suggested a high level of corrosion resistance exhibited by EAD.

#### 3.6.2. Alternating Current Impedance Spectra

**Figure 4:**
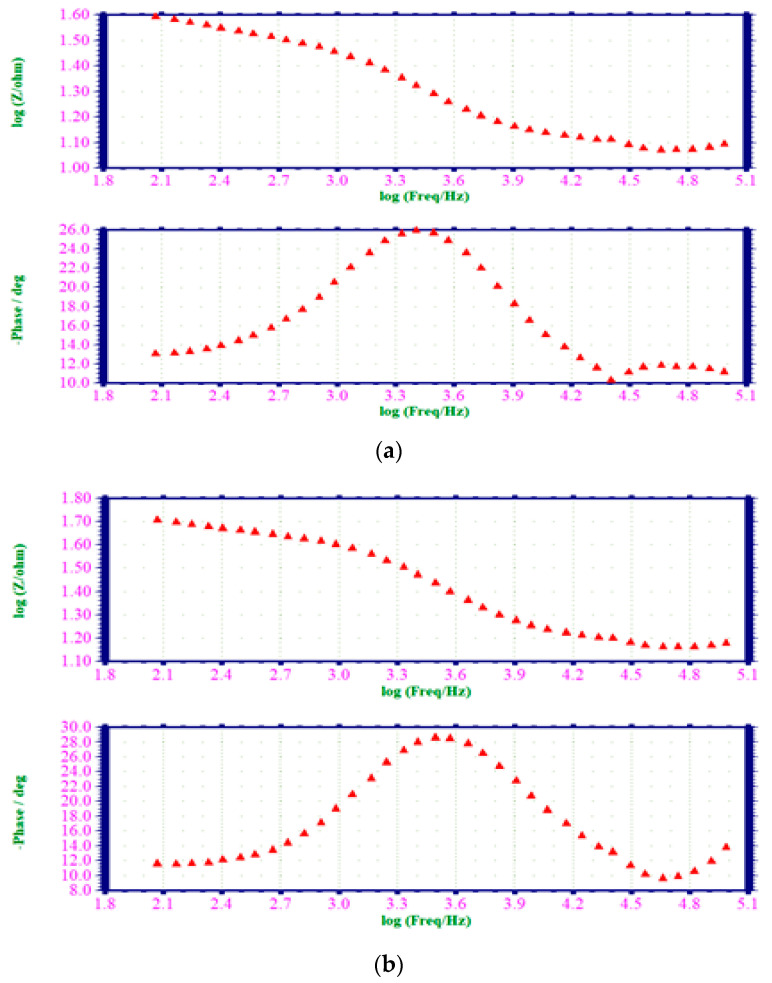
(**a**) AC impedance spectra of mild steel dipped in 1 M HCl (Bode Plot); (**b**) AC impedance spectra of specimen dipped in 1 M HCl with 0.001 M of EAD inhibitor (Bode Plot).

The use of electrochemical impedance spectra has been employed to confirm the formation of a protective coating on the surface of mild steel [46]. The formation of a protective coating on the surface of mild steel has been reported to increase the charge transfer resistance (*R_t_*). Simultaneously, there was a decrease in the double-layer capacitance (*C_dl_*), although the logarithm of impedance (Z/ohm) showed an increase. Figure 4 presented the AC impedance spectra, specifically the Nyquist plots, of mild steel when it was immersed in a hydrochloric acid solution with a concentration of 1 M. The spectra were shown for two scenarios: one without the presence of an EAD inhibitor system and the other with the inclusion of an inhibitor. Additionally, Figure 4a,b displayed the Bode plots corresponding to the same experimental conditions. The impedance spectra of mild steel, which were subjected to immersion in several test solutions, were precisely measured within the realm of alternating current. The values of *R_t_* and *C_dl_* were presented in Table 6.

When mild steel was exposed to HCl solution with a concentration of 1 M, the resulting *R_t_* was found to be 23.62 ohm cm^2^, and the corresponding *C_dl_* value was 42.3779 × 10^−6^ F cm^−2^. The *R_t_* exhibited an increase from 23.62 to 35.44 ohm cm^2^ after introducing a 0.001 M concentration of EAD into a 1 M HCl blank solution. The *C_dl_* value exhibited a decrease from 42.3779 × 10^−6^ to 57.6733 × 10^−6^ F cm^−2^. The impedance value (Z/ohm) experienced an increase from 0.502 to 0.532 in its logarithmic form. In addition, it was noticed that the phase angle of the inhibitor system displayed an increase from 25.9 degree to 28.4 degree compared to the blank [47]. This discovery indicated the presence of a protective coating formation on the surface of the mild steel substrate.

### 3.7. Surface Analysis of Mild Steel by SEM

**Figure 5:**
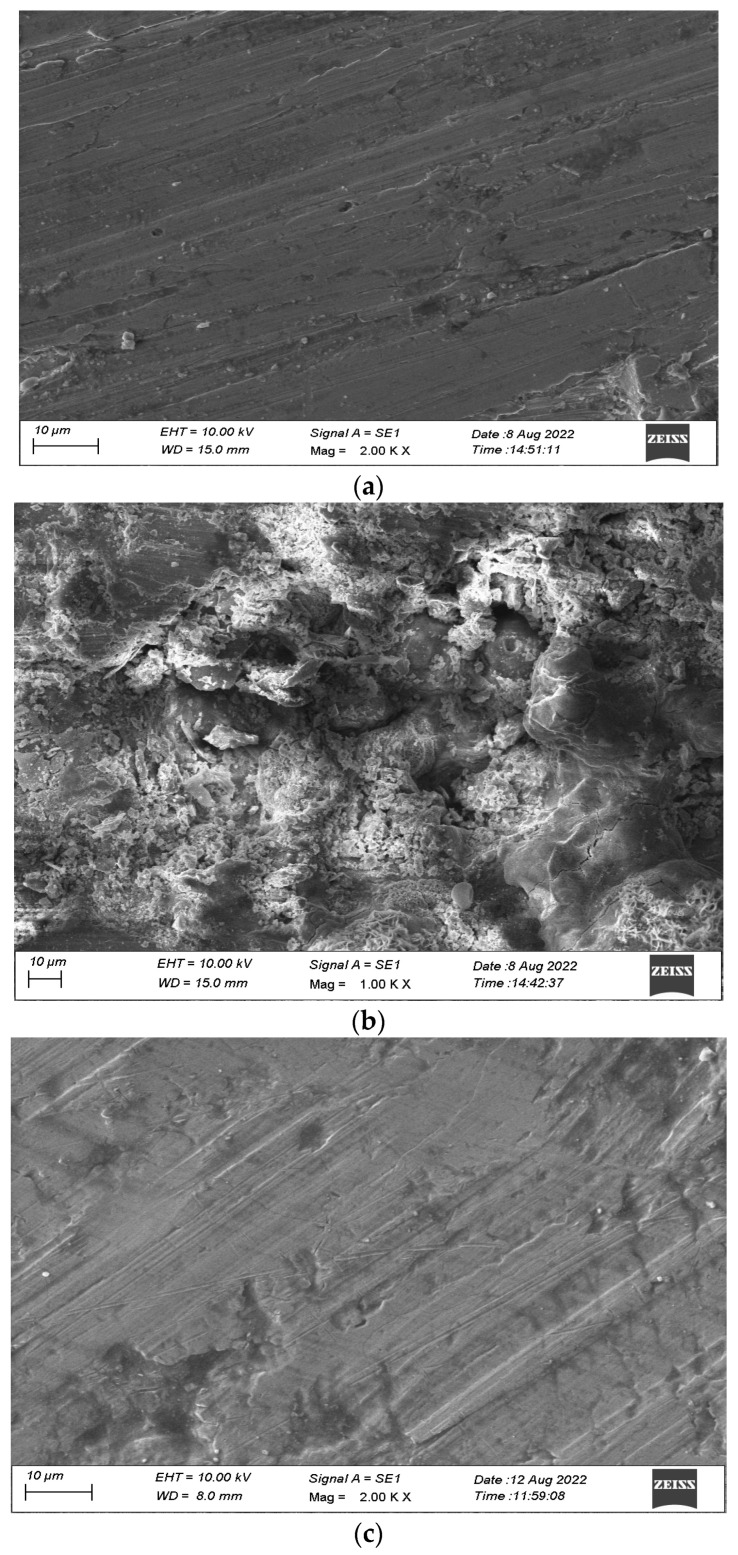
(**a**) SEM image of polished mild steel specimen (control); (**b**) SEM image of mild steel coupon after immersion in 1 M HCl (blank); (**c**) SEM image of polished specimen coupon after immersion in 1 M HCl with 0.001 M of EAD.

The utilization of scanning electron microscopy enables the elucidation of surface morphology, thereby providing valuable insights into the composition and structure of surface coatings applied to the mild steel. This investigation ascertains the magnitude of corrosion and its occurrence in the presence and absence of corrosion inhibitors by performing a comprehensive examination of the surface morphology of mild steel through the utilization of SEM images, both in the presence and absence of inhibitor [48,49], to comprehend the characteristics of the coating on the surface with and without EAD inhibitor, and to assess the deterioration of the mild steel. Figure 5a–c depicted the visual representation of the specimen immersed in a solution of HCl with a concentration of 1 M for 2 h, with both the occurrence and non-occurrence of an electrochemical acid dissolution (EAD) inhibitor system.

**Figure 6:**
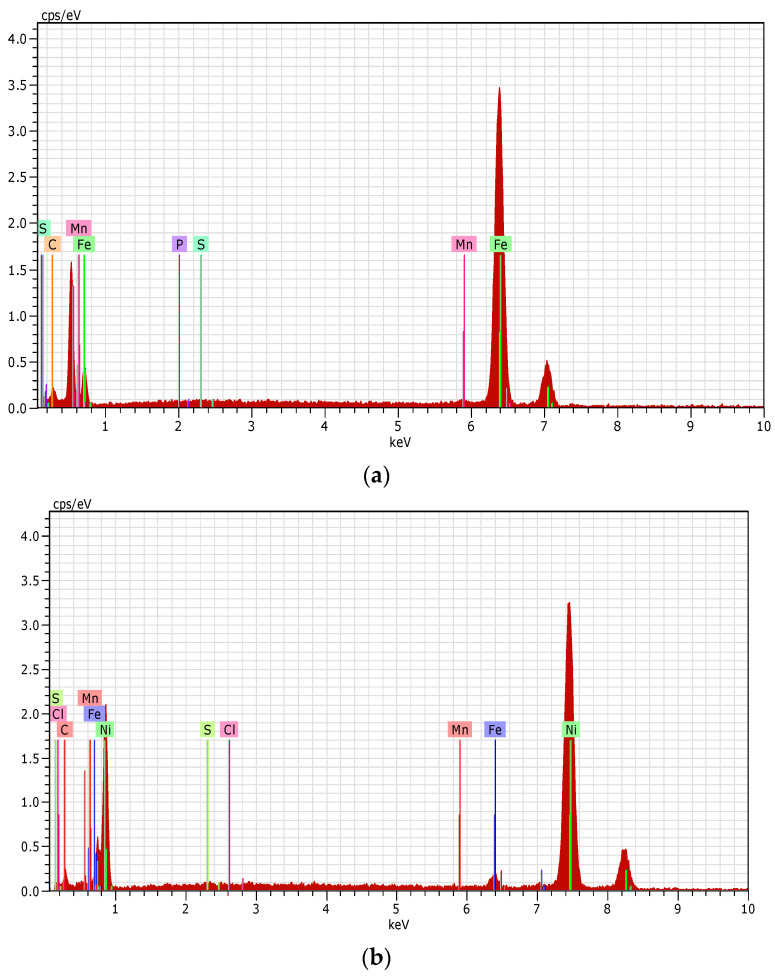

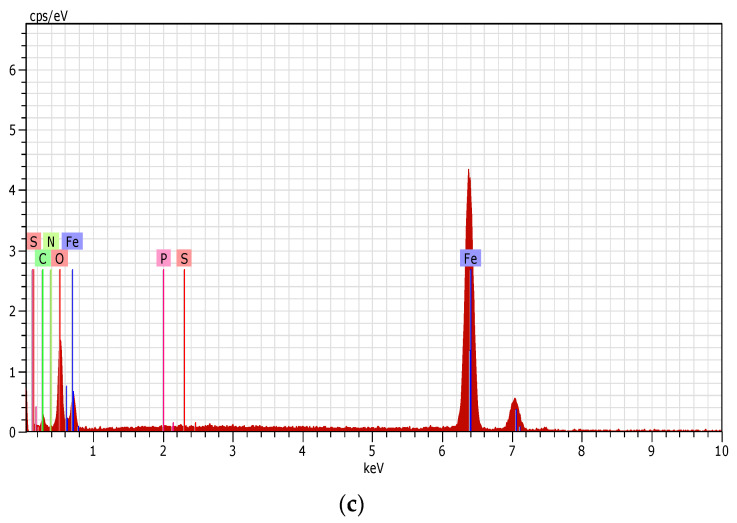
(**a**) EDAX spectrum of mild steel specimen (control); (**b**) EDAX spectrum of mild steel specimen immersed in 1 M HCl (blank); (**c**) EDAX spectrum of mild steel specimen immersed in 1 M HCl with 0.001 M of EAD.

Figure 5a (control) exhibited a polished mild steel specimen characterized by a pristine, unblemished surface devoid of any discernible formation of corrosion inhibitors. Figure 5b exhibits the manifestation of moderate steel surface corrosion resulting from the dissolution of the metal in an HCl solution with a concentration of 1 M. The surface exhibits a granular texture akin to that of mild steel, indicative of the occurrence of localized corrosion. Figure 6c depicted the surface of a specimen immersed in a solution of HCl with a concentration of 1 M, wherein an inhibitor was present. The corrosion inhibition was observed as a reduction in the surface area affected by corrosion upon the addition of the amiodarone drug at a concentration of 0.001 M. The precipitation of an insoluble compound on mild steel mitigated the occurrence of corrosion. The carbon steel specimen exhibited a surface texture akin to that of meticulously polished mild steel [50,51]. A monolayer of inhibitor on the substrate effectively regulated the attrition in an HCl solution with a concentration of 1 M.

### 3.8. Surface Characterization of Mild Steel via Energy Dispersive Analysis (EDAX)

The EDAX spectra of steel corroded in the presence and absence of EAD were performed to analyze element composition on the surface of mild steel, and the corresponding spectrum were given in Figure 6. This spectrum showed the mild steel’s constituents have diminished in concentration. In addition, the presence of Cl^−^ indicated the corrosion of mild steel following contact with a 1 M HCl solution (Figure 6c). The effect observed was consistent with a weakening of chlorine signals and a strengthening of signals from surface components. The incidence of an inhibitor accounts for the emergence of the Fe signal and the subsequent amplification of the O signal. All of this information points to iron (Fe), sulfur (S), carbon (C), phosphorous (P), nickel (Ni), nitrogen (N), and oxygen (O) atoms on the surface of the carbon steel. The presence of an inhibitor in the corrosive system was responsible for the creation of this layer. The inhibitor dramatically reduced the intensity of the Fe peaks, as seen in Figure 6c compared to the blank solution of 1 M HCl. This finding points to the existence of an adsorbed inhibitor layer, which protects mild steel against rust. These results suggested that the oxygen, nitrogen, and carbon atoms at a concentration of 0.001 M of EAD may undergo adsorption on the surface of the steel sample. As a result, a Fe^2+^ EAD was formed on the surface to protect steel from corrosion [52,53,54,55].

### 3.9. Surface Characterization of Mild Steel via Atomic Force Microscopy

**Figure 7:**
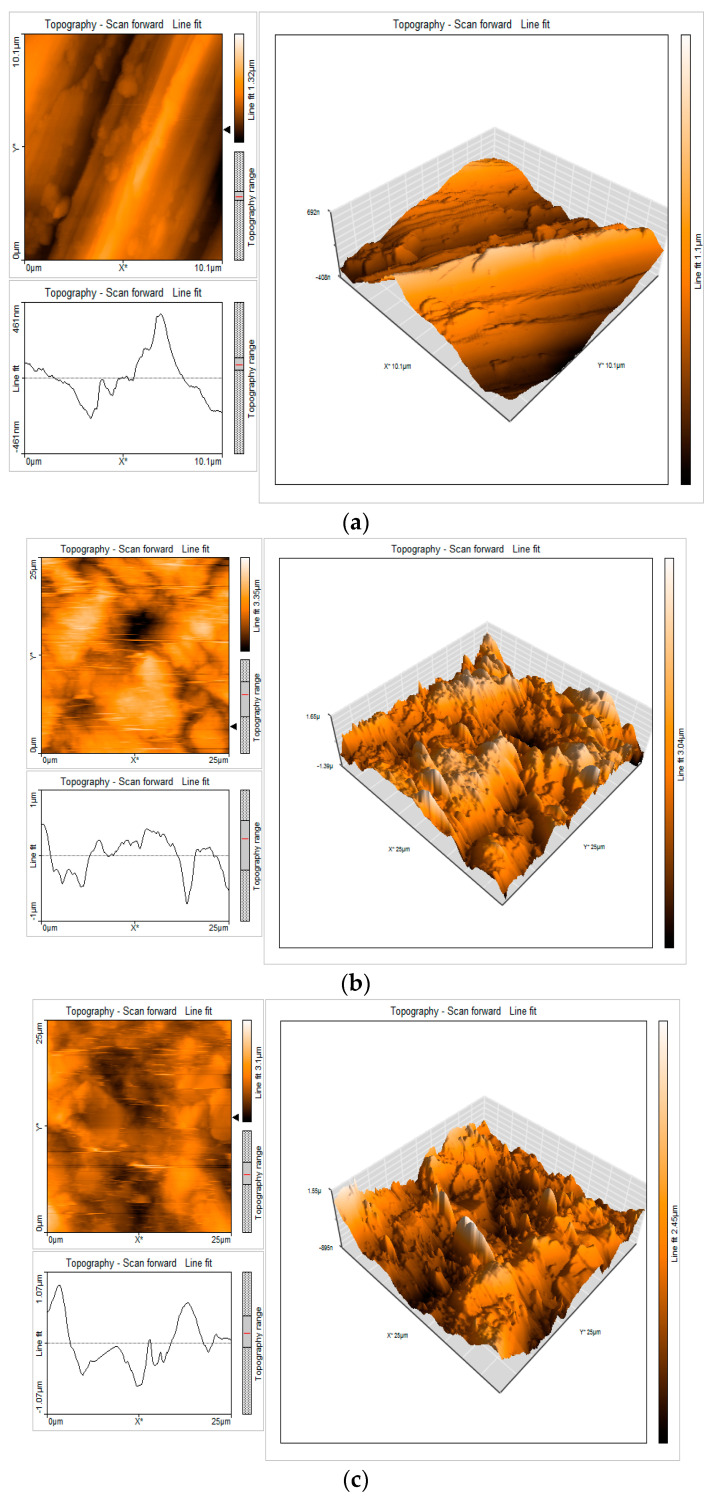
(**a**) AFM cross-sectional image of the polished sample surface (control); (**b**) AFM cross-sectional image of the surface of the sample after immersion in 1 M HCl (blank); (**c**) AFM cross-sectional image of the surface after immersion in 1 M HCl with 0.001 M of EAD inhibitor.

Atomic force microscopy (AFM) was an indispensable technique for the acquisition of surface roughness data about various mild steel surfaces. The exploration of the surface morphology of mild steels entails the scrutiny of the existence of coated protective layers utilizing AFM [56]. The examination of surface roughness can be conveniently conducted via AFM investigations. It provides a direct and illuminating viewpoint on modifications transpiring within the surface morphology at the nano-scale level. Following the occurrence of surface topography modifications in mild steel as a consequence of corrosion and the subsequent formation of a safeguarding stratum, it was discerned that these alterations manifest dissimilarly in the nonexistence and existence of inhibitors. The given depiction illustrates the portrayal of three-dimensional (3D) AFM structures and the AFM cross-sectional depiction of the polished sample surface (control sample), the sample in a 1 M hydrochloric acid solution (blank sample), and the surface treated with 0.001 M of EAD while immersed in a 1 M HCl solution. These visual representations can be observed in Figure 7a–c.

The examination of AFM images was carried out to ascertain the surface roughness parameters. These parameters encompass the average surface roughness, *S_a_*, which signifies the mean deviation of all points on the roughness profile from a reference line across the assessment span. The root-mean-square surface roughness, denoted as *S_q_*, was determined by taking the average of the measured height deviations observed within the evaluation length, with respect to the mean line. The *S_y_* value, representing the maximum peak-to-valley height, was determined as the larger of the two values. The atomic force microscopy (AFM) parameters were elucidated in Table 7.

These parameters encompass the average surface roughness (*R_a_*), which quantifies the average displacement of all points on the roughness profile from a reference line across the specified evaluation distance. Furthermore, the root-mean-square surface roughness, denoted as *R_q_*, was determined by averaging the measured height deviations observed within the evaluation length in relation to the mean line. The atomic force microscopy (AFM) parameters were presented in Table 8.

The average surface roughness of carbon steel in a medium (blank) exhibited a significantly elevated magnitude. In the presence of a 0.001 M concentration of EAD inhibitor, this value was reduced drastically. The aforementioned value exhibited a lower magnitude compared to the corrosive medium (undisclosed), while manifesting a higher magnitude relative to the polished surface. The observed phenomenon can be explained by the presence of a 0.001 M concentration of an EAD inhibitor, which leads to the creation of a protective film on the outermost layer of the material. This film exhibited a high degree of surface uniformity. Similarly, the aforementioned scenario applied to the remaining three parameters, namely root mean square roughness, maximum peak to valley height, and maximum peak height [57,58,59,60].

## 4. Conclusions

The comprehensive investigation into the corrosion inhibition potential of expired and unused pharmaceutical EAD (amiodarone) for mild steel in a hydrochloric acid (HCl) solution yielded quantifiable data across various analytical techniques. The weight loss method revealed a remarkable corrosion inhibition efficiency (IE) of 88.77% at a concentration of 0.001 M EAD. The temperature-dependent corrosion rate and IE data underscored the significance of temperature in influencing the inhibitor’s performance, with IE decreasing from 88.77% to 34.70% as the temperature increased from 303 K to 333 K.

The adsorption isotherm analysis, specifically using the Temkin adsorption isotherm, provided quantifiable data on the adsorption energy, further elucidating the molecular interactions between EAD and the mild steel surface. The potentiodynamic polarization studies quantified the corrosion potential shift and changes in corrosion parameters, indicating the cathode inhibition behavior of EAD. Electrochemical impedance measurements quantified the increase in charge transfer resistance (*R_t_*) and the decrease in double-layer capacitance (*C_dl_*), confirming the formation of a protective coating on the mild steel surface.

Surface analyses through SEM, EDAX, and AFM provided visual and elemental quantifications of the corrosion and inhibition processes. SEM images quantifiably depicted the reduction in the extent of corrosion on the mild steel surface in the presence of EAD. EDAX spectra quantified the elemental composition, and AFM data provided numerical values for surface roughness parameters, showcasing a significant reduction in roughness in the presence of EAD.

## Figures and Tables

**Table 1:** Effect of EAD concentration on CR and IE.

Concentration of EAD Inhibitor (M)	Corrosion Rate (mm y^−1^)	Inhibition Efficiency (%)	Surface Coverage (θ)
-	0.8386	-	-
0.0000001	0.3508	58.16	0.5816
0.000001	0.2738	67.35	0.6735
0.00001	0.2225	73.46	0.7346
0.0001	0.1540	81.63	0.8163
0.001	0.0941	88.77	0.8877

**Table 2:** Effect of temperature on CR and IE.

Temperature (K)	Corrosion Rate (mm y^−1^)	Inhibition Efficiency (%)	Surface Coverage (θ)
303	0.0941	88.77	0.8877
308	0.1198	85.71	0.8571
313	0.1711	79.59	0.7959
318	0.2481	70.41	0.7041
323	0.3594	57.14	0.5714
328	0.4621	44.89	0.4489
333	0.5476	34.70	0.3470

**Table 3:** Average free energy of adsorption of specimen at various temperatures in 1 M HCl in the presence of EAD.

Inhibitor System	${-\Delta G}_{ads}^{o}$ (kJ/mol^−1^)						
	303 K	308 K	313 K	318 K	323 K	328 K	333 K
1 M HCl + EAD inhibitor	26.9287	26.6642	25.9733	25.0781	23.9208	22.9536	22.1205

**Table 4:** The value of *E_a_* for blank and EAD inhibitor.

System	*E_a_* (KJ/mol)
Blank	20.127
EAD inhibitor	52.3865

**Table 5:** Potentiodynamic Polarization parameters for the decomposition of specimen in 1 M HCl without and with of EAD inhibitor system.

Concentration of the EAD Inhibitor (M)	*E_corr_* mV/SCE	Tafel Slope		*I_corr_* A/cm^2^	LPR Ω/cm^2^
		ba mV/dec	bc mV/dec		
Blank	−632	0.152	0.208	1.2730 × 10^−4^	291
0.001	−672	0.110	0.322	4.6340 × 10^−4^	792

**Table 6:** Electrochemical impedance parameters from Nyquist plots for the corrosion of mild steel submerged in 1 M HCl in the absence and presence of EAD.

Concentration of the EAD Inhibitor (M)	Nyquist Plot		Impedance Log (Z/ohm)	Phase Angle (Degree)
	$R_{t}\;\Omega$/cm^2^	*C_dl_* F/cm^2^		
-	23.69	42.3779 × 10^−6^	0.502	25.9
0.001	35.44	57.6733 × 10^−6^	0.532	28.4

**Table 7:** AFM data: area roughness.

Samples	*S_a_* (nm)	*S_q_* (nm)	*S_y_* (nm)	*S_p_* (nm)
Mild steel surface	136.47	168.68	892.96	476.88
Mild steel surface immersed in 1 M HCl	379.59	476.21	3295.40	1530.6
Mild steel surface immersed in 1 M HCl + 0.001 M of EAD	274.51	344.64	2639.30	1679.00

**Table 8:** AFM data: line roughness.

Samples	*R_a_* (nm)	*R_q_* (nm)	*R_y_* (nm)	*R_p_* (nm)
Mild steel surface	119.44	161.1	684.87	406.66
Mild steel surface immersed in 1 M HCl	344.72	393.18	1359.9	726.51
Mild steel surface immersed in 1 M HCl + 0.001 M of EAD	198.54	256.62	1099.60	662.49

## Data Availability

Data are contained within the article.
